# Mindfulness Meditation for Sleep Disturbances Among Individuals with Cognitive Impairment: A Scoping Review

**DOI:** 10.3390/healthcare13030296

**Published:** 2025-01-31

**Authors:** Sunny H. W. Chan, Richard Cheston, Charlotte Steward-Anderson, Chong-Ho Yu, Emily Dodd, Elizabeth Coulthard

**Affiliations:** 1 Centre for Health and Clinical Research, University of the West of England, Bristol BS16 1QY, UK; 2 School of Social Sciences, University of the West of England, Bristol BS16 1QY, UK; richard.cheston@uwe.ac.uk; 3 School of Health and Social Wellbeing, University of the West of England, Bristol BS16 1QY, UK; c.anderson26@nhs.net (C.S.-A.); emily3.dodd@uwe.ac.uk (E.D.); 4 College of Natural and Computational Sciences, Hawaii Pacific University, Honolulu, HI 96813, USA; cayu@hpu.edu; 5 Bristol Medical School, University of Bristol, Bristol BS8 1QU, UK; elizabeth.coulthard@bristol.ac.uk

**Keywords:** mindfulness meditation, sleep disturbances, dementia, mild cognitive impairment (MCI), non-pharmacological interventions

## Abstract

**Background**: This scoping review investigates the effectiveness of mindfulness meditation in alleviating sleep disturbances among individuals with mild cognitive impairment (MCI) and Alzheimer’s disease (AD). With the rising prevalence of dementia and its profound impact on cognitive function and quality of life, this review aims to synthesize existing research and identify gaps in the literature. **Methods**: We systematically searched six electronic databases (CINAHL, Embase, Medline, PsycINFO, PubMed, and Scopus) from 2004 to 2024, yielding 462 potentially relevant articles. Screening was conducted using ASReview, an AI ranking tool, which facilitated the selection of studies. Ultimately, seven studies that met our stringent eligibility criteria were included in the review. We adhered to the Preferred Reporting Items for Systematic Reviews and Meta-Analyses extension for Scoping Reviews (PRISMA-ScR) guidelines for reporting. **Results**: Our findings indicate that mindfulness meditation significantly improves sleep quality, reduces insomnia severity, and enhances overall well-being in this at-risk population. Notably, interventions that combine structured, face-to-face sessions with at-home practice emerged as the most effective. **Conclusions**: Despite these positive outcomes, methodological limitations, including small sample sizes and reliance on self-reported measures, underscore the need for more rigorous long-term studies. This review highlights the potential of mindfulness meditation as a low-cost, scalable intervention to improve sleep and cognitive health in older adults, paving the way for future research and clinical applications.

## 1. Introduction

Dementia is a significant health concern that is increasingly affecting populations worldwide as they age. Currently, around 50 million people worldwide are living with dementia, a figure expected to rise to 78 million by 2030 and reach 152 million by 2050 [1,2]. This increase is largely attributed to an aging population, with projections indicating significant future rises in dementia prevalence. Since 1990, the total number of individuals affected by dementia has more than doubled, driven by population growth and demographic changes [3]. It profoundly impacts both individuals with dementia and their caregivers, leading to a notable decline in quality of life [4]. Currently, there are limited treatment options for dementia, which makes it a relevant issue in healthcare [5]. Dementia is a broad term that encompasses a range of symptoms indicating a decline in memory, language skills, problem-solving abilities, and other cognitive functions, which ultimately affects an individual’s daily functioning. AD is the leading cause of dementia [6]. The AD continuum consists of three primary phases: preclinical AD, MCI due to AD, and dementia due to AD [7,8,9].

Sleep disturbances are common throughout the AD continuum, affecting both individuals at risk of developing dementia and those already diagnosed with the condition [10]. A recent systematic review [11] found that the pooled prevalence of any symptoms of sleep disturbance among individuals with dementia was 26% (95% confidence interval [CI]: 23–30%; n = 2719). Additionally, the prevalence of clinically significant sleep disturbance was reported at 19% (95% CI: 13–25%; n = 2753). These disturbances can lead to a range of negative consequences. Poor sleep can result in fatigue, which exacerbates cognitive decline and impairs daily functioning [12]. Additionally, sleep deprivation often contributes to mood disturbances, further impacting emotional well-being. Disrupted sleep patterns can also create significant strain on caregivers, complicating the caregiving environment [13,14]. Moreover, unresolved sleep issues can lead to behavioral disturbances, such as agitation and aggression, which pose challenges for care provision and may increase the risk of early institutionalization [15,16].

Sleep disruption in individuals with dementia is closely linked to the pathophysiology of AD [17,18]. Adverse factors such as anxiety, depression, stress, and sleep disturbances elevate the risk of developing dementia. Sleep disorders are potential risk factors for cognitive impairment [19]. Specifically, a bidirectional and causal relationship between non-rapid eye movement (NREM) sleep and amyloid beta pathophysiology may contribute to both the risk and progression of AD [20,21]. Research indicates a significant relationship between beta-amyloid deposition and atrophy occurring early in the disease process of AD [22], and addressing modifiable risk factors during the early stages of neurodegeneration when impairment is relatively mild, is a promising approach to prolong independence and good quality of life [23]. Approximately one-third of dementia cases worldwide may be influenced by modifiable factors, underscoring the high potential impact of preventive strategies [24].

The primary approach for treating sleep disorders in individuals with MCI or AD often begins with pharmacological interventions, including sedative hypnotics such as benzodiazepines and unregulated supplements like melatonin [25]. However, pharmacotherapy has notable limitations, including potential side effects, drug interactions, and the risk of dependence. These medications can expose patients to various harms, such as increased sedation, falls, and further cognitive decline [26]. In response to these concerns, there has been a rise in psychological and behavioral therapies for sleep disorders in recent decades. A notable trend is the growing interest among older adults in exploring alternative therapies, such as mind–body interventions, as options for addressing sleep issues [27,28,29,30]. However, the evidence supporting many of these alternative approaches remains inconclusive.

Mindfulness meditation is a mind–body intervention that encompasses various strategies aimed at regulating emotions and attention, ultimately promoting overall well-being and emotional balance [31]. This practice typically includes both guided sessions with an instructor and daily home practice. Research has shown that meditation can positively impact cognitive functions, such as attention and memory, as well as improve health and well-being in the aging population [32,33].

The effectiveness of mindfulness meditation in addressing sleep problems is well-documented [34,35,36]. Specifically, mindfulness meditation can help individuals cope with insomnia by fostering acceptance of their sleep difficulties, reducing sleep pressure, minimizing adverse brain stimulation, and promoting better sleep [37]. From a biological standpoint, mindfulness training has been shown to enhance neural protection by targeting neuron-restrictive silencing factors in individuals with MCI and AD [38]. As a result, it is not surprising that mindfulness meditation has been linked to a decreased risk and delayed onset of dementia [39,40]. Nevertheless, a recent systematic review [41] and meta-analyses [42,43] underscore that the application of mindfulness-based intervention for addressing sleep issues in individuals with MCI or dementia remains limited. There is a pressing need to enhance study methodologies, as this is vital for advancing our understanding of the mechanisms underlying mindfulness-based interventions for patients with MCI and AD, as well as their long-term effects.

Recently, several systematic reviews and meta-analyses have examined non-pharmacological interventions aimed at alleviating sleep disturbances in individuals with MCI or AD [44,45,46,47]. However, these reviews have primarily focused on interventions such as light therapy, electrotherapy stimulation, physical exercise, acupressure/acupuncture, massage, cognitive behavioral therapy for insomnia (CBT-I), and other multi-modal approaches. Notably, there has been a lack of studies exploring the impact of mindfulness meditation, with only one systematic review [46] including just two relevant studies on this topic. While CBT-I is typically the first-line treatment for insomnia, a recent study utilizing micro-costing has demonstrated that mindfulness-based interventions are more cost-effective [48]. This approach not only offers significant savings for both caregivers and the healthcare system but also effectively addresses insomnia. This gap highlights the need for a systematic scoping review to assess the effectiveness of mindfulness meditation in addressing sleep problems among individuals with MCI or AD. Additionally, there is limited evidence regarding the implementation of mindfulness meditation to improve sleep in this population. A clearer understanding of how such interventions can be designed and executed to enhance sleep quality in individuals with MCI or AD is essential.

## 2. Materials and Methods

The research on mindfulness interventions and their effect on sleep in older individuals with MCI or AD is still in its early stages. The existing literature in this field has not been consolidated to validate any substantial findings. This review aimed to outline the methods and content of these interventions, along with their impacts; therefore, a scoping review is the most suitable approach for consolidating the research evidence [49]. It is particularly valuable when addressing broad research questions and when recommendations for future research are required [50]. This scoping review followed the Joanna Briggs Institute (JBI) guidelines for scoping reviews [51,52] and PRISMA-ScR guidelines to consolidate findings [53]. The review protocol was registered in PROSPERO (CRD42024528961).

### 2.1. Search Strategy

We systematically searched six electronic databases from 2004 to 2024: CINAHL, Embase, Medline, PsycINFO, PubMed, and Scopus. Randomized or non-randomized clinical trials in which mindfulness meditation was compared to control conditions in people with MCI or AD were included. The selection criteria were based on the Population, Intervention, Comparison, Outcome (PICO) model. Studies that met the following criteria (Table 1) were included in the scoping review.

### 2.2. Eligibility Critiera

To be included in this review, articles needed to be peer-reviewed, written in English, and report either observational (i.e., pre/post design) or controlled studies (non-randomized and randomized trials). Studies were excluded if they had no available full text or were gray literature.

### 2.3. Study Selection

In our systematic review, we conducted comprehensive searches across multiple scientific databases, which yielded 462 potentially relevant articles. To eliminate duplicates, we utilized reference manager EndNote X9 and review manager Covidence. However, due to EndNote’s limited sensitivity in identifying duplicates, we performed additional deduplication in Covidence [54], resulting in a deduplicated set of articles. All types of randomized controlled trials, observational studies, case–control studies, or cross-sectional studies were considered eligible for inclusion.

For screening the titles and abstracts, the first author employed the AI tool ’ASReview’ (Version 0.17.1) [55]. This tool utilizes an active researcher-in-the-loop machine learning algorithm to rank articles based on their probability of meeting the inclusion criteria through text mining. The AI tool presents the top-ranked article to the reviewer, who makes the decision to include or exclude it for full-text screening. This decision is then factored into the subsequent ranking, and the next top-ranked article is proposed to the reviewer, employing an active learning approach. In particular, five references that met the inclusion criteria were selected along with five irrelevant references randomly suggested by the program for training purposes. It is important to note that the AI tool suggests articles based on their probability of relevance, but it is ultimately the human reviewer who decides which articles to include or exclude. An explanation of how the AI-supported screening was implemented and the decision-making process for this review has been reported in detail by van Dijk et al. [56].

**Figure 1:**
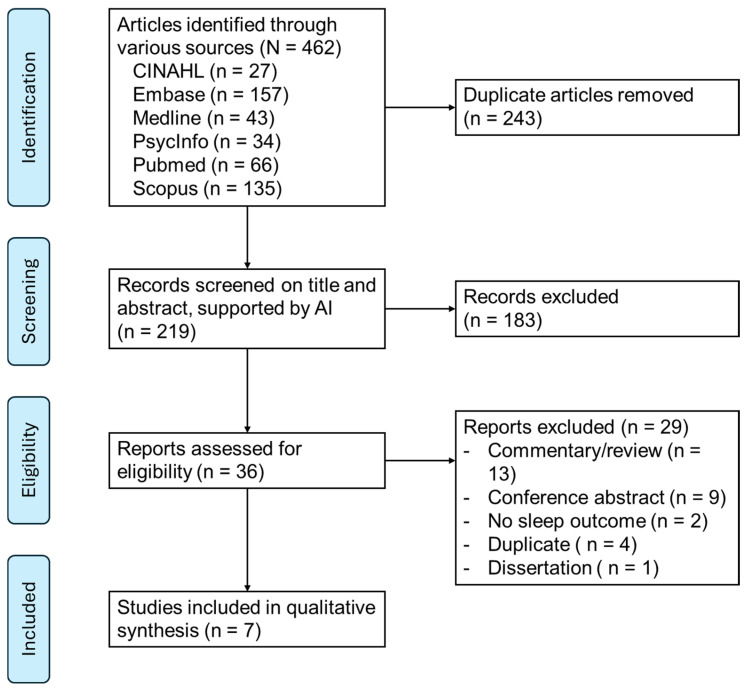
PRISMA flow diagram.

To minimize the impact of subjectivity on inclusion, the articles identified as relevant during the title and abstract screening underwent independent full-text screening by two reviewers. In cases of disagreement regarding inclusion, a third independent reviewer was consulted to resolve the discrepancies. The PRISMA-ScR flow diagram [53] summarizes the disposition of all articles identified (Figure 1).

### 2.4. Data Extraxction and Synthesis

The data from all included studies were extracted and checked using a standard data extraction form. The following information was recorded in our review: study design, participants characteristics, intervention details, sleep outcomes, other outcomes, participation, and adherence level. Each article was extracted independently by two authors, and consensus was reached on the data extraction for each article.

### 2.5. Critical Appraisal

Critical appraisal is a systematic process for evaluating research studies, playing a vital role in evidence-based practice. It involves scrutinizing various aspects of a study to assess the reliability and validity of its outcomes, which is essential for determining the credibility of interventions in healthcare research. All studies included in this scoping review were critically appraised using the Mixed Methods Appraisal Tool (MMAT) Version 2018 [57]. The MMAT is a distinctive tool designed to evaluate empirical qualitative, quantitative, and mixed-method study designs. It consists of five specific questions tailored to each study design. Researchers rate each component of the MMAT as ‘Yes’, ‘No’, or ‘Can’t tell’, with guidance provided to assist in reaching a comprehensive conclusion.

## 3. Results

### 3.1. Study Identification

The search yielded 219 unique articles (Figure 1). After 183 exclusions in the title and abstract review phase, full-text review was conducted on 36 articles. After excluding 29 articles due to various reasons, such as commentary or conference abstract, seven articles were reviewed and synthesized (Table 2).

### 3.2. Participant Characteristics

Three studies focused on individuals with subjective cognitive decline, utilizing evidence-based criteria from recent prospective research [58,59,60]. Cai et al. [61] examined individuals with MCI, while Giulietti et al. [62] targeted participants in the early stages of AD, requiring formal diagnoses for inclusion. These studies implemented strict exclusion criteria, eliminating participants with various medical conditions, neurological and psychiatric diagnoses, and specific medications.

In contrast, Paller et al. [63] and Kovach et al. [64] employed broader eligibility criteria, accepting patients with varying levels of cognitive impairment not exclusively related to AD. Kovach et al. [64] excluded only individuals with movement disorders, while Paller et al. [63] did not impose exclusions based on other medical conditions.

The selected studies focus on participants aged 50 to 98 years, with mean ages varying from 60.47 years to 87 years. Female representation is notably high, with percentages ranging from 59.5% to 86.79%. This demonstrates a significant predominance of female participants in the research on cognitive health across these age groups (Table 2).

**Table 2:** Characteristics of participants and study settings.

Author et al. (Year)	Participants—Total N	Age Range (Mean ± sd) in Years	Female %	Health Conditions	Country	Recruitment
Innes et al. (2016) [60]	N = 60	50–84 (60.6 ± 1.0) years	85%	Subjective cognitive decline, Metabolic/vascular risk	USA	Community health and workplace settings
Innes et al. (2021) [58]	N = 40	50–84 (64.2 ± 1.4) years	72%	Subjective cognitive decline, multiple health issues	USA	Community settings via flyers
Innes et al. (2018) [59]	N = 60	50–84 (60.47 ± 1.17) years	86.79%	Subjective cognitive decline; 94% had at least one metabolic/vascular risk factor for AD	USA	Healthcare, community, and workplace settings
Paller et al. (2015) [63]	N = 37	55–81 (72) years caregivers: 31–98 (62.5)	59.5%	Various cognitive deficits	USA	University Alzheimer’s Disease Center, local advertisements
Kovach et al. (2018) [64]	N = 36	56–98 (87 ± 10.2) years	80.56%	Various chronic illnesses, cognitive impairment	USA	Nursing homes and assisted living settings
Giulietti et al. (2023) [62]	N = 90	>70 (82.8 ± 5.6) years	63.6%	Early-stage Alzheimer’s disease	Italy	Neurology clinic
Cai et al. (2022) [61]	N = 75	60+ (80 ± 9.3) years	74.7%	Mild Cognitive Impairment, sleep disturbances	China	Nursing homes via flyers and postings

### 3.3. Intervention Characteristics

All studies mandated some form of face-to-face intervention delivery (Table 3). Three studies included an initial in-person training session, after which participants carried out the intervention at home [58,59,60]. Notably, Innes et al. [58] replicated their design [60], maintaining the same intervention structure but adding a control group.

Three studies utilized a hybrid approach, combining weekly face-to-face sessions with assigned “homework” tasks that participants completed independently or with caregiver support. These tasks were typically related to the most recent session and encouraged participants to integrate mindfulness practices into their daily lives [61,62,63]. Five out of the seven studies required participants to engage in daily practice lasting between 10 and 45 min [58,59,60,61,63]. Kovach et al. [64] was the only study to conduct mindfulness practice in a group setting.

Session lengths averaged from 45 min [64] to 1.5 h [61], with program durations varying widely from 4 weeks [64] to 6 months [62]. Interventions were led by mindfulness teachers [61,64] and psychotherapists [62], while facilitators in other studies were not specified.

The content of the programs varied, including activities such as music listening [60], Mindfulness-Based Stress Reduction (MBSR) with gentle yoga and breathwork [62,63], compassion meditation [64], and intersensory practices like Kirtan Kriya Meditation, which involves breathwork, finger movements (mudras), mantra, and visualization [60]. Paller et al. [63] also incorporated elements from dialectical behavior therapy and acceptance and commitment therapy.

### 3.4. Participation and Adherence

All studies meticulously tracked participant involvement and compliance (Table 3). However, three studies [59,61,63] did not provide detailed explanations for all dropouts. Notably, Paller et al. [63] reported a dropout rate of 16%, while Innes et al. [59] had a slightly higher rate of 21%.

The impact of the COVID-19 pandemic was acknowledged in two studies. Giulietti et al. [62] faced challenges in delivering interventions to their control group of 22 participants, originally targeting a sample size of 80, due to losing 36 participants during lockdown. Similarly, Cai et al. [61] encountered difficulties in collecting follow-up data at 3, 6, and 12 months but maintained a strong retention rate, with only a 5% dropout rate among their sample of 75 participants.

Kovach et al. [64] documented the lowest retention rate, with only 28% of the active intervention group attending all four weeks of sessions. They noted that 11% of participants were non-participatory, while 33% struggled to understand or follow instructions during the ’Present in the Now’ mindfulness intervention sessions.

Innes et al. [58,60] conducted a comprehensive analysis of participant engagement by comparing retention, adherence, and treatment expectations across both studies, revealing minimal discrepancies. Innes et al. [60] demonstrated a robust retention rate of 92% for the 12-week intervention and 88% for the full 6-month duration, while the 2021 study maintained an overall retention rate of 80%.

### 3.5. Sleep Outcomes

All studies utilized self-report tools to evaluate sleep outcomes, as detailed in Table 3. Six out of seven studies employed the global score of the Pittsburgh Sleep Quality Index (PSQI) to assess sleep quality. Additionally, Cai et al. [61] aimed to gauge insomnia severity using the Insomnia Severity Index (ISI) and the Athens Insomnia Scale (AIS).

Three studies also incorporated objective measures of sleep. Innes et al. [59] examined plasma Aβ levels, a biomarker of cognitive decline linked to sleep quality. Cai et al. [61] utilized electroencephalography (EEG) readings to analyze changes between mindful and resting states. Kovach et al. [64] employed a sleep actigraphy wrist device to measure various parameters, including total sleep time, sleep efficiency, wake after sleep onset, and the sleep fragmentation index.

Interestingly, Giulietti et al. [62] did not include a specific measure for sleep quality. Instead, they used the SF-36 [65], a tool for assessing health-related quality of life, which includes domains such as ’energy fatigue’ and ’physical functioning’. They also utilized the Neuropsychiatric Inventory (NPI) to screen for nocturnal behavior disorders.

### 3.6. Other Outcomes

All studies assessed various additional outcomes (Table 3), including memory, psychomotor speed, attention, and executive function, primarily relying on self-report tools. Cognitive functions were evaluated across multiple studies, emphasizing memory and other domains such as psychomotor speed, attention, and executive function [58,59,60,61].

Psychiatric outcomes measured included stress [58,59,60,64], depression [62,63], agitation [64], psychological well-being, psychosocial aspects, spiritual well-being, and quality of life [58,59,60,63]. Notably, only Kovach et al. [64] employed objective measures beyond self-reports, analyzing participants’ salivary cortisol levels as biomarkers of stress.

Additionally, two studies incorporated feedback from caregivers, albeit with different focuses. Giulietti et al. [62] sought information solely about the patients from their caregivers, while Paller et al. [63] considered both the patients’ conditions and the health and well-being of the caregivers themselves.

### 3.7. Mechanisms of Mindfulness on Sleep “Intervention Effects”

The mindfulness interventions explored in the reviewed studies were primarily linked to positive changes in sleep quality and energy levels (Table 3). Innes et al. [58,60] investigated Kirtan Kriya (KK) meditation, which involves repeated mantra chanting, visualization, and hand movements (mudras), and compared it with a music listening control group. Results indicated that both groups experienced improvements in stress, mood, well-being, sleep, and quality of life (QOL), with particularly pronounced benefits in the KK group, sustained at the six-month mark. This may be attributed to the meditation practice’s ability to reduce stress and promote relaxation, key factors in enhancing sleep and alleviating fatigue.

Innes et al. [59] further demonstrated that KK meditation not only improved sleep quality but also positively correlated with plasma Aβ levels, suggesting a potential bidirectional relationship between enhanced sleep and reductions in Alzheimer’s disease-related biomarkers. This study underscored the role of mindfulness in alleviating cognitive fatigue and enhancing memory function and mood.

Giulietti et al. [62] found that a six-month mindfulness-based intervention reduced fatigue in early-stage Alzheimer’s patients, leading to fewer sleep disturbances and nighttime behavior disorders. Improvements in neuropsychiatric symptoms, such as anxiety and agitation, may have contributed to better nighttime rest and daytime energy levels. Similarly, Cai et al. [61] reported that an eight-week mindfulness meditation course, combined with daily practice, resulted in significant reductions in insomnia severity and improvements in sleep efficiency. These changes were supported by both subjective measures (PSQI, Athens Insomnia Scale) and objective measures (EEG), likely contributing to increased overall energy and reduced fatigue.

Kovach et al. [64] was the only study involving participants with severe cognitive decline, utilizing a crossover design with both the ‘Present in the Now’ (PIN) mindfulness program and a control group engaged in ‘Cognitive Therapeutic Activity’ (COG) over 11 sessions in four weeks. Preintervention findings indicated that participants had prolonged bedtimes and fragmented sleep. Although no changes were observed in nocturnal sleep measures for either group, PIN participants reduced their daytime napping by an average of 27 min daily. While the study did not provide insights into sleep quality beyond the one-week follow-up, the reduction in napping may foster healthier sleep hygiene and routines, potentially creating a cumulative positive impact over time. This research offers valuable insights for implementing mindfulness practices in residential settings to mitigate risk behaviors associated with institutionalization.

Paller et al. [63] made a unique observation that caregivers reported poorer sleep quality than patients. Both groups experienced a similar reduction in sleep problems, with 40% of participants noting improvements. Among those who initially reported sleep issues, two-thirds experienced improvement. This study highlights the feasibility of conducting mindfulness interventions for mixed groups of caregivers and patients, demonstrating cost-effective methods to address the unmet needs of caregivers. Overall, the studies provide strong evidence that mindfulness interventions positively influence sleep by enhancing self-awareness, reducing stress, promoting relaxation, and mitigating fatigue, thereby improving both physical and mental energy levels.

### 3.8. Critical Appraisal

All studies included in this scoping review utilized a group-based design and were critically appraised using the MMAT [57]. Results are presented in Table 4.

#### 3.8.1. Randomized Control Trials

The synthesis of these studies highlighted several strengths and limitations in study design. Six out of seven studies were critically appraised as randomized controlled trials (RCTs). Of these, four reported the randomization method appropriately. Specifically, all studies by Innes et al. [58,59,60] utilized a randomly varying block randomization method. Kovach et al. [64] conducted a controlled crossover repeated measures experimental study, which randomized the order in which participants received the active and control interventions.

Two studies [61,62] indicated that randomization was used but did not provide details regarding the quality of the randomization methods employed. Five out of six studies reported baseline demographics and scores, with four indicating that the groups were primarily comparable at baseline. One study did not outline baseline characteristic data; however, the crossover design employed by Kovach et al. [64] mitigates the potential influence of confounding variables.

Outcome assessors were blinded to the interventions in four out of six studies [58,59,60,61], and participants adhered to the assigned intervention in those studies. In contrast, Kovach et al. [64] did not blind the outcome assessors, and only 28% of participants attended all sessions, indicating low adherence. Both this study and Cai et al. [61] utilized Intention to Treat (ITT) analysis, considered the ‘gold standard’ for interpreting RCTs, as it reduces bias and supports prognostic balance [66]. Giulietti et al. [62] did not mention blinding, and the control group was not treated according to their assigned intervention.

#### 3.8.2. Quantitative Non-Randomized Trials

Paller et al. [63] utilized a quasi-experimental pre-post study design. In the critical appraisal using the MMAT, the study participants were deemed representative of the target population, as the authors clearly defined the target population and applied inclusion and exclusion criteria that aligned with it. Given that this was a feasibility pilot study aimed at laying the groundwork for future RCTs, the measurements for both outcomes and interventions were appropriate, with no deviations from the proposed design noted.

However, the completion of outcome data remains unclear, and six dropouts were not adequately explained. While the exclusion criteria were not detailed, suggesting the potential presence of confounding variables, the discussion section thoughtfully addresses the influence of these confounders to avoid overinterpreting the internal validity of the study.

## 4. Discussion

This study was undertaken to (1) describe and synthesize the evidence base on mindfulness meditation to improve sleep among people with cognitive impairment, an area of inquiry that has not previously been undertaken, and (2) to identify gaps in the published evidence to guide potential avenues for future intervention work. The synthesis of evidence from the reviewed articles underscores a growing body of research supporting mindfulness interventions for enhancing overall well-being, particularly sleep, in older adults, especially those with MCI and related conditions. The effectiveness of these interventions indicates that mindfulness can serve as a low-cost, scalable therapy that can be easily integrated into care settings. Various studies have examined different types of mindfulness interventions, measurement techniques, and reported effectiveness, while also acknowledging methodological weaknesses and proposing avenues for future research.

### 4.1. Insights for Enhancing Minfulness Inerventions for Sleep

The findings from the reviewed studies highlight the diverse approaches to mindfulness interventions aimed at improving sleep, emphasizing the significance of structured, face-to-face delivery combined with at-home practices. Looking ahead, several directions for further research and implementation of mindfulness in sleep enhancement can be considered. Firstly, there is a pressing need for long-term studies to evaluate the sustained effects of mindfulness interventions on sleep quality. Gaining insight into how these practices influence sleep over extended periods can help establish their efficacy and inform best practices [67]. Secondly, the variety of approaches suggests the potential for integrating mindfulness with other therapeutic methods—such as cognitive behavioral therapy for insomnia (CBT-I) [68], tai chi [27], or innovative technology [69]—to create synergistic effects and offer more comprehensive treatment options. To enhance adherence to mindfulness interventions, various strategies can be explored. For instance, utilizing mobile apps and online platforms [70,71] could broaden access to mindfulness practices. Incorporating features such as guided sessions, reminders, and progress tracking may further encourage adherence and support participants in maintaining their routines.

The feasibility of applying mindfulness meditation in individuals with severe dementia raises significant concerns, particularly given the challenges associated with maintaining attentional focus over extended periods. The high dropout rate observed in one of the reviewed studies [64], with 44% of participants unable to follow the program, suggests that traditional mindfulness-based interventions may not be suitable for this demographic without modifications. For individuals with severe dementia, adaptations are essential to accommodate their cognitive limitations. This may involve shorter sessions, more frequent breaks, and the use of simpler techniques that require less sustained attention. By tailoring mindfulness practices to meet the specific needs of this population, we can enhance engagement and potentially improve outcomes, making mindfulness a more viable option for individuals facing cognitive challenges. As such, the applicability of this non-pharmacological intervention is highly dependent on the stage of the disease.

### 4.2. Enhancing Validity in Sleep Outcome Measures

Standardized measures for assessing sleep outcomes, such as PSQI, are valuable which can enhance comparability across studies and facilitate future meta-analyses, ultimately strengthening the evidence base for mindfulness interventions aimed at improving sleep. However, the reliance on self-report tools raises concerns about subjectivity and recall bias, particularly when applied to individuals with cognitive impairment [72]. Consequently, high rates of inconsistent PSQI responses are often observed among individuals with cognitive impairment [73]. To enhance the validity of findings, future studies could benefit from a more balanced approach that combines subjective measures with objective assessments. The reviewed studies highlight that incorporating objective measures, such as EEG readings and actigraphy, offers a more nuanced understanding of sleep dynamics. It would be valuable to discuss the implications of these findings, particularly in relation to how they correlate with self-reported outcomes. Future research could delve into the relationship between subjective perceptions of sleep quality and objective sleep data, providing deeper insights into the effectiveness of mindfulness interventions.

### 4.3. Possible Mechanisms of Mindfulness Interention in Sleep

In addition to the primary sleep outcomes, nearly all the reviewed studies employed various outcome measures. The focus on multiple cognitive functions—such as memory, psychomotor speed, attention, and executive function—suggests that mindfulness interventions may offer broader cognitive benefits beyond just improving sleep. Enhancements in these cognitive domains could indirectly lead to better sleep quality. Moreover, the inclusion of mental health-related measures, such as stress, depression, and agitation, indicates that mindfulness practices may address underlying mental health issues that impact sleep. This provides valuable insights into the mechanisms through which mindfulness exerts its effects. Notably, stress reduction, emotional regulation, and improved cognitive functioning could serve as key mediators in the relationship between mindfulness and sleep. These insights can deepen our understanding of how mindfulness interventions work and facilitate the development of more targeted and effective strategies for enhancing sleep quality.

### 4.4. Methodological Limitations

Despite the promising findings, the limitations of the included studies reveal several common issues that could affect the reliability and generalizability of their results. Small sample sizes and a lack of long-term follow-up data significantly constrain the applicability of the findings [62]. For instance, Kovach et al. [64] noted that their small sample size limits broader applicability, compounded by a two-week washout period that may have introduced carryover effects. Additionally, the data collector was not blinded to the interventions, which could introduce bias in observational measures, and the reliance on objective measures rather than self-reports may have further impacted outcomes. Similarly, Paller et al. [63] encountered challenges with sample size and diversity, raising questions about whether the observed benefits can be solely attributed to mindfulness. Innes et al. [60] faced comparable limitations, including a small, well-educated sample and the absence of diagnostic cognitive testing, potentially overlooking undiagnosed MCI. This study also lacked a control group for usual care. Cai et al. [61] was limited to nursing home residents, which may skew the results, and follow-up assessments were affected by the COVID-19 pandemic. Giulietti et al. [62] also had a small sample size and was conducted at a single institution, restricting the generalizability of its findings to the broader Alzheimer’s disease population. Innes et al. [59] primarily included well-educated, motivated participants and did not assess episodic memory, while the lack of blinding in treatment administration could introduce additional bias. Lastly, Innes et al. [58] faced challenges related to small sample sizes and self-selection bias, which may not adequately represent the wider population experiencing subjective cognitive decline.

Furthermore, many studies relied on self-reported measures, which can introduce bias and compromise the reliability of outcomes. Overall, these studies exhibit limitations related to sample size, generalizability, the absence of control groups, and potential biases, underscoring the necessity for cautious interpretation of their findings. Addressing these weaknesses in future research is essential for strengthening the evidence base for mindfulness as an intervention for older adults with cognitive impairments.

### 4.5. Future Directions

Future research should prioritize the development of larger, well-controlled trials that incorporate diverse populations and settings. Randomized controlled trials comparing mindfulness against other interventions, such as cognitive behavioral therapy or physical exercise, could elucidate the unique contributions of mindfulness practices. Additionally, longitudinal studies assessing the long-term impacts of mindfulness on cognitive function and sleep quality would provide valuable insights into its efficacy over time. Investigating the underlying neurophysiological mechanisms of mindfulness through advanced imaging techniques could further enhance our understanding of its benefits for cognitive health.

## 5. Conclusions

In summary, the existing scoping review presents notable evidence supporting the effectiveness of mindfulness interventions for enhancing sleep quality and cognitive function in older adults, particularly those with MCI. While methodological limitations persist, future research is poised to expand our understanding of how mindfulness can be integrated into care models for aging populations. Through rigorous studies and diverse interventions, mindfulness may prove to be a transformative approach in promoting cognitive health and overall well-being among older adults.

## Figures and Tables

**Table 1:** Full search criteria.

P (Population)	Dementia or “mild cognitive impairment” or “Alzheimer’s disease” or “Frontotemporal dementia” or “Lewy-body dementia” or “vascular dementia” or “mixed dementia” or “subjective cognitive decline” or “memory decline” or “memory loss”.
I (Intervention)	Mindfulness or meditation.
C (Comparison)	Standard therapy or no treatment.
O (Outcome)	Sleep or awakening or wake or wakefulness or sleepiness or nap or doze or insomnia.

**Table 3:** Study design and intervention participation.

Author et al. (Year)	Study Design Type Follow Up Period	Intervention Description Active Comparator	Outcomes Sleep Others	Main Findings Sleep Others	Participation and Adherence
Innes et al. (2016) [60]	RCT Six months	KK meditation Format: involves a multifaceted exercise that includes chanting a mantra, performing a mudra, and visualization techniques Duration: 12 weeks Frequency: Daily Session length: 12 min Content: includes the repetition of the ’Sa-Ta-Na-Ma’ mantra while engaging in specific finger movements (mudra) and visualizations related to sound energy entering and exiting the body 2. Music listening Format: Participants listened to a selection of relaxing instrumental music from various composers Duration: 12 weeks Frequency: daily Session length: 12 min Content: The program CD included music from six composers. Participants were encouraged to choose their musical selections	1. PSQI 2a. Cognition Subjective: Memory function Questionnaire (MFQ) Executive function (Trail-Making Test—TMT) Psychomotor speed and attention and working memory (90-second Wechsler Digit Symbol Substitution Test—DSST) 2b. Psychosocial and QOL Subjective: Perceived Stress Scale (PSS) 65 profile of mood states (POMS) Psychological Well-being Scale (PBWS) Health-related QOL (SF-36)	Both groups demonstrated significant improvements in sleep quality. The KK group showed a greater effect in terms of sleep quality compared to the ML group. Both KK meditation and ML can lead to improvements in various psychosocial outcomes with more pronounced benefits from KK meditation.	Retention: 92% of participants (27/30 in the KK group and 28/30 in the ML group completed the 12-week intervention. 88% (26/30 in KK and 27/30 in ML) completed the full 6-month study period. Dropout Reasons: Included family emergencies, time constraints, and being lost to follow-up. Adherence Rates: Participants completed an average of 93% of the 84 possible sessions during the first 12 weeks. During the optional 3-month follow-up period, adherence was 71%.
Innes et al. (2021) [58]	Randomized feasibility trial Three months	1. KK meditation Ditto 2a. Music listening Ditto 2b. Enhance usual care Format: Includes a comprehensive, illustrated educational booklet regarding healthy aging and dementia. Brain health activities. Duration: 12 weeks Frequency: daily Session length: 12 min Content: Covers general information on aging, memory loss, dementia risk factors, strategies for healthy aging, medication management, and resources for additional information, support, and volunteer opportunities	Ditto	Both the active treatment (KK and ML) and enhanced usual care (EUC) groups showed improvements in sleep quality over time Both KK and ML led to significant improvements in mood and perceived memory functioning, and quality of life compared to the EUC group.	Retention: 80% participants completed the 3-month intervention. EUC has a much better retention. Dropout Reasons: Personal illness or family emergencies, conflicts with religious beliefs, other conflicts, and lost to follow-up Adherence Rates: 84.4% of participants remaining in the study submitting completed daily logs.
Innes et al. (2018) [59]	Exploratory randomized clinical trial Six months	KK meditation Ditto Music listening Ditto	1a. Subjective: PSQI 1b. Objective: Blood biomarkers (telomere length (TL), telomerase activity (TA), and plasma amyloid-β (Aβ) levels) associated with sleep quality 2a. Cognition Ditto 2b. Psychosocial and QOL Ditto	KK group demonstrated significantly greater increases in plasma Aβ40 levels compared to the ML group (*p* = 0.04) Both groups showed significant improvements in memory function and cognitive performance at 3 and 6 months. Improvements in the KK group were greater in perceived stress, mood, and QOL-mental health compared to the ML group	Retention: 48 out of the 53 participants (91%) completing the 12-week intervention program. Furthermore, 47 participants (89%) completed the full 6-month study, indicating a strong retention rate. Dropout reasons: Did not specify particular reasons for dropout Adherence rates: Participants completing an average of 94% of the sessions during the 12-week intervention period (93% in the KK group and 95% in the ML group). During the optional 3-month follow-up period, adherence was slightly lower, with participants completing an average of 71% of the sessions (68% for KK and 74% for ML)
Paller et al. (2015) [63]	Pre-post intervention design Two weeks	Mindfulness training program Format: Weekly group sessions (both patients and caregivers participate together) Duration: Eight weeks Frequency: Once a week Content: Sessions included progression of mindfulness practice such as attending to breathing, bodily sensations, movement and thoughts and acceptance. Homework related to weekly sessions	PSQI 2a. Quality of life in AD (QOL-AD) Depression Scale (GDS) Trail-Making Tests A and B 2b. Carer distress regarding patient problems as measured by Revised Memory Problem and Behaviour Checklist (RMPBC)	Among participants who initially reported sleep problems, there was a significant improvement of 1.5 points (F(1, 22) = 4.72, P = 0.041) 2a. Participants experienced an average increase of 1.8 points in Quality of Life ratings (QOL-AD) The average score on the Geriatric Depression Scale (GDS) decreased by 1.4 points (F(1, 35) = 4.16, *p* = 0.049 improvements on the Trail-Making Test Part B, indicating enhanced cognitive control and task switching (F(1,2 3) = 11.11, *p* = 0.03 2b. Caregivers showed a trend toward decreased distress regarding patient problem	Retention: Out of the initial participants, six individuals dropped out before completing the procedure, indicating a dropout rate of approximately 16.2%. Dropout reasons: Do not specify particular reasons for dropout Adherence rates: 71% of participants reported using mindfulness techniques regularly after the program. Additionally, 84% felt they benefited from the program, and 89% indicated they would recommend it to others. These high rates of perceived benefit and willingness to continue mindfulness practices suggest that adherence to the program’s teachings was relatively strong among those who completed it
Kovach et al. (2018) [64]	Controlled crossover repeated measures experimental design One week, and then underwent 2-week washout period	“Present in the Now” (PIN) mindfulness intervention Format: Conducted in a group setting. It is designed to be pragmatic and foster emotional well-being, with an emphasis on continued practice Duration: 45 min for each session Frequency: Involves 11 sessions, which are held mid- to late-morning on 2 days in the first week and 3 days per week for the next 3 weeks Content: Three main components—attentional skill exercises, body awareness activities, and compassion meditation. 2. Cognitive therapeutic activity (COG) intervention Format: Group-based cognitive activities that stimulated memory and thinking Duration: 45 min for each session Frequency: Involves 11 sessions, which are held mid- to late-morning on 2 days in the first week and 3 days per week for the next 3 weeks Content: Included cognitive activities such as wordplay, mental aerobics, and trivia. These activities were designed to stimulate cognitive engagement without the focus on mindfulness or emotional regulation	Sleep actigraphy wrist device. Measuring total sleep time, sleep efficiency, wake after sleep onset and sleep fragmentation index (restlessness) Subjective: Agitation—Cohen-Mansfield Agitation Inventory; Affect—Observed Emotion Rating Scale; Engagement—Arousal states in Dementia Scale; Interoception and Discomfort— Dementia of the Alzheimers type scale. Communication of need report Objective: Stress—Salivary Cortisol Assay	No changes in nocturnal sleep in the PIN and COG groups for any of the measures PIN participants decreased their daytime napping duration by an average of 27 min. PIN intervention led to short-term decreases in agitation and discomfort compared to COG control group	Retention: 29 out of 36 participants (81%) attended seven or more of the 11 sessions offered for the PIN intervention, and 11 participants (28%) attended all sessions. In contrast, in the COG group, only 18 participants (50%) attended seven or more sessions. Dropout reasons: Three individuals from the PIN group dropped out within the first week, citing that the activity was not what they expected and did not wish to continue. Additionally, one participant never received the COG intervention due to hospitalization and extended rehabilitation Adherence rates: No specified
Giulietti et al. (2023) [62]	Randomized controlled trial Six months	1. Mindfulness-based intervention Format: Weekly group sessions led by a single psychotherapist with specific training in mindfulness and extensive meditation experience Duration: Six months Frequency: 1 h session each week Content: The first month focuses on learning stress management exercises, specifically the Jacobson relaxation technique, which involves practicing relaxation for 1.5 min three times a week. After the initial month, participants begin meditative practices associated with MBIs, exercising for 15–20 min three times a week (two times at home and once in the therapeutic setting), while continuing relaxation training. 2. No intervention Standard care or cognitive training as per usual practice.	Neuropsychiatric inventory—sleep and nighttime behavior disorders Everyday Cognition Scale–ECOG; Quality of life by SF-36; Spiritual well-being (SWB); Depression by Beck depression inventory (BDI); Neuropsychiatric inventory MMSE.	MBI showed a reduction in sleep and nighttime behavior disorders after six months of treatment (*p* < 0.005). In contrast, the untreated patients experienced a worsening of sleep and nighttime behavior disorders during the same period For the MBI group, significant improvement in ECOG, all domains in SF-36, SWB, BDI, and neuropsychiatric symptoms. No decline in cognitive status as no change in MMSE	Retention: Not specified Dropout reasons: Not specified Adherence rates: Not specified
Cai et al. (2022) [61]	Double-blind parallel randomized controlled trial No follow-up due to COVID-19 pandemic	Mindfulness therapy Format: Structured sessions led by an experienced instructor. Each session involved mindfulness practices such as mindful awareness, breathing exercises, and body scans. Duration: Eight weeks Frequency: Once a week for 1.5 h per session Content: Each session included various mindfulness practices and themes, such as mindful breathing, body scan, or mindful stretching. To support daily practice, audio recordings of each session were provided to participants, and nursing home staff organized the mindfulness practice at a fixed time and place each day. 2. Health education group Format: Structured educational sessions led by a trained geriatric nurse Duration: Eight weeks. Frequency: Once a week for 1.5 h per session. Content: Each session included various topics aimed at improving sleep and cognition. The sessions covered biological characteristics of sleep, sleep and cognition, self-monitoring of sleep, mild cognitive impairment interventions, and cognitive training in daily life.	PSQI Insomnia Severity Index (ISI); Athens Insomnia Scale (AIS) EEG—interpreting changes during the mindful state versus the rest state. 2a. Cognitive domains: Language, memory, executive function, attention. 2b. Psychological well-being: Depression, anxiety, stress.	The PSQI, ISI, and AIS evaluations demonstrated a decrease in insomnia severity in the intervention group when compared to the control group, supported by EEG findings. 2a. Significant improvement in cognitive domains in mindfulness therapy group. 2b. Significant reduction in anxiety and stress in mindfulness therapy group.	Retention: Not specified Dropout reasons: Not specified Adherence rates: Not specified

**Table 4:** Appraisal of studies.

For Randomized Control Trials (RCTs)						
Author et al. (Year)	Type of Study	2.1. Is Randomisation Appropriately Performed?	2.2. Are the Groups Comparable at Baseline?	2.3. Are There Complete Outcome Data?	2.4. Are Outcome Assessors Blinded to the Intervention Provided?	2.5 Did the Participants Adhere to the Assigned Intervention?
Innes et al. (2016) [60]	RCT—2 arms	YES	YES	YES	YES	YES
Innes et al. (2021) [58]	Randomized feasibility trial—three arms	YES	YES	YES	YES	YES
Innes et al. (2018) [59]	Exploratory randomized clinical trial	YES	YES	YES	YES	YES
Kovach et al. (2018) [64]	Controlled crossover repeated measures experimental design	YES	YES	YES	NO The data collector was not blinded to study arm	SOMEWHAT Only 28% of participants attended all sessions
Giulietti et al. (2023) [62]	RCT with two arms	YES	YES	YES	NO It did not mention blinding	YES
Cai et al. (2022) [61]	Double-blind parallel RCT	YES	YES	YES	YES	YES
**For Quantitative Non-Randomized Studies**						
**Author et al. (year)**	**Type of Study**	**3.1. Are the Participants Representative of the Target Population?**	**3.2. Are Measurements Appropriate Regarding both the Outcome and Intervention (or Exposure)?**	**3.3. Are There Complete Outcome Data?**	**3.4. Are the Confounders Accounted for in the Design and Analysis?**	**3.5 During the Study Period, Is the Intervention Administered (or Exposure Occurred) as Intended?**
Paller et al. (2015) [63]	Pre-post intervention design Quasi-experimental	YES	YES	NO Did not specify the completeness of outcome data	NO It lacks a control group for direct comparison, which may limit the ability to fully account for confounders	YES

## Data Availability

The data supporting the findings of this study are available from the corresponding author upon reasonable request.

## References

[1] Patterson C. (2018). World Alzheimer Report 2018.

[2] World Health Organization (2021). Global Status Report on the Public Health Response to Dementia.

[3] Nichols E., Szoeke C.E.I., Vollset S.E., Abbasi N., Aichour M.T.E., Akinyemi R.O., Asgedom S.W., Awasthi A., Barker-Collo S.L., Baune B.T. (2019). Global, regional, and national burden of Alzheimer’s disease and other dementias, 1990–2016: A systematic analysis for the Global Burden of Disease Study 2016. Lancet Neurol..

[4] Sikkes S.A.M., Tang Y., Jutten R.J., Wesselman L.M.P., Turkstra L.S., Brodaty H., Clare L., Cassidy-Eagle E., Cox K.L., Chételat G. (2021). Toward a theory-based specification of non-pharmacological treatments in aging and dementia: Focused reviews and methodological recommendations. Alzheimer’s Dement. J. Alzheimer’s Assoc..

[5] Alzheimer’s Association (2015). 2015 Alzheimer’s disease facts and figures. Alzheimer’s Dement..

[6] Porsteinsson A.P., Isaacson R.S., Knox S., Sabbagh M.N., Rubino I. (2021). Diagnosis of Early Alzheimer’s Disease: Clinical Practice in 2021. J. Prev. Alzheimer’s Dis..

[7] Albert M.S., DeKosky S.T., Dickson D., Dubois B., Feldman H.H., Fox N.C., Gamst A., Holtzman D.M., Jagust W.J., Petersen R.C. (2011). The diagnosis of mild cognitive impairment due to Alzheimer’s disease: Recommendations from the National Institute on Aging-Alzheimer’s Association workgroups on diagnostic guidelines for Alzheimer’s disease. Alzheimer’s Dement..

[8] McKhann G.M., Knopman D.S., Chertkow H., Hyman B.T., Jack C.R., Kawas C.H., Klunk W.E., Koroshetz W.J., Manly J.J., Mayeux R. (2011). The diagnosis of dementia due to Alzheimer’s disease: Recommendations from the National Institute on Aging-Alzheimer’s Association workgroups on diagnostic guidelines for Alzheimer’s disease. Alzheimer’s Dement..

[9] Sperling R.A., Aisen P.S., Beckett L.A., Bennett D.A., Craft S., Fagan A.M., Iwatsubo T., Jack C.R., Kaye J., Montine T.J. (2011). Toward defining the preclinical stages of Alzheimer’s disease: Recommendations from the National Institute on Aging-Alzheimer’s Association workgroups on diagnostic guidelines for Alzheimer’s disease. Alzheimer’s Dement..

[10] Greene L., Aryankhesal A., Megson M., Blake J., Wong G., Briscoe S., Hilton A., Killett A., Reeve J., Allan L. (2022). Understanding primary care diagnosis and management of sleep disturbance for people with dementia or mild cognitive impairment: A realist review protocol. BMJ Open.

[11] Koren T., Fisher E., Webster L., Livingston G., Rapaport P. (2023). Prevalence of sleep disturbances in people with dementia living in the community: A systematic review and meta-analysis. Ageing Res. Rev..

[12] Tan X., Åkerstedt T., Lagerros Y.T., Åkerstedt A.M., Bellocco R., Adami H.-O., Ye W., Pei J.-J., Wang H.-X. (2023). Interactive association between insomnia symptoms and sleep duration for the risk of dementia—A prospective study in the Swedish National March Cohort. Age Ageing.

[13] Liang J., Aranda M.P., Lloyd D.A. (2020). Association between Role Overload and Sleep Disturbance among Dementia Caregivers: The Impact of Social Support and Social Engagement. J. Aging Health.

[14] Simón M.A., Bueno A.M., Blanco V., Otero P., Vázquez F.L. (2022). Sleep disturbance, psychological distress and perceived burden in female family caregivers of dependent patients with dementia: A case-control study. Healthcare.

[15] van Duinen-van den I.J.C.L., Mulders A., Smalbrugge M., Zwijsen S.A., Appelhof B., Zuidema S.U., de Vugt M.E., Verhey F.R.J., Bakker C., Koopmans R. (2018). Nursing staff distress associated with neuropsychiatric symptoms in young-onset dementia and late-onset dementia. J. Am. Med. Dir. Assoc..

[16] Kim S.-S., Oh K.M., Richards K. (2014). Sleep disturbance, nocturnal agitation behaviors, and medical comorbidity in older adults with dementia: Relationship to reported caregiver burden. Res. Gerontol. Nurs..

[17] Hahn E.A., Wang H.X., Andel R., Fratiglioni L. (2014). A change in sleep pattern may predict Alzheimer disease. Am. J. Geriatr. Psychiatry.

[18] Ju Y.E., McLeland J.S., Toedebusch C.D., Xiong C., Fagan A.M., Duntley S.P., Morris J.C., Holtzman D.M. (2013). Sleep quality and preclinical Alzheimer disease. JAMA Neurol..

[19] da Silva R.A.P.C. (2015). Sleep disturbances and mild cognitive impairment: A review. Sleep Sci..

[20] Mander B.A., Winer J.R., Jagust W.J., Walker M.P. (2016). Sleep: A Novel Mechanistic Pathway, Biomarker, and Treatment Target in the Pathology of Alzheimer’s Disease?. Trends Neurosci..

[21] Mander B.A. (2013). Disturbed sleep in preclinical cognitive impairment: Cause and effect?. Sleep.

[22] Chételat G., Villemagne V.L., Bourgeat P., Pike K.E., Jones G., Ames D., Ellis K.A., Szoeke C., Martins R.N., O’Keefe G.J. (2010). Relationship between atrophy and beta-amyloid deposition in Alzheimer disease. Ann. Neurol..

[23] Livingston G., Sommerlad A., Orgeta V., Costafreda S.G., Huntley J., Ames D., Ballard C., Banerjee S., Burns A., Cohen-Mansfield J. (2017). Dementia prevention, intervention, and care. Lancet.

[24] Norton S.P., Matthews F.E.P., Barnes D.E.P., Yaffe K.P., Brayne C.P. (2014). Potential for primary prevention of Alzheimer’s disease: An analysis of population-based data. Lancet Neurol..

[25] Xu J., Wang L.-L., Dammer E.B., Li C.-B., Xu G., Chen S.-D., Wang G. (2015). Melatonin for sleep disorders and cognition in dementia: A meta-analysis of randomized controlled trials. Am. J. Alzheimer’s Dis. Other Dement..

[26] Salami O., Lyketsos C., Rao V. (2011). Treatment of sleep disturbance in Alzheimer’s dementia. Int. J. Geriatr. Psychiatry.

[27] Chan S.H.W., Ng S.M., Yu C.H., Chan C.M., Wang S.M., Chan W.C. (2022). The effects of an integrated mindfulness-based tai chi chuan programme on sleep disturbance among community-dwelling elderly people: Protocol for a randomized controlled trial. Trials.

[28] Kligler B.M.D.M.P.H., Teets R.M.D., Quick M.D.O. (2016). Complementary/Integrative Therapies That Work: A Review of the Evidence. Am. Fam. Physician.

[29] Liu J., Yang Y., Li C., Perez A., Raine A., Shi H., Zou L. (2024). Effects of Mind-Body Qigong Exercise on Overall Health, Fatigue/Sleep, and Cognition in Older Chinese Immigrants in the US: An Intervention Study with Control. J. Aging Res..

[30] Voiß P., Höxtermann M.D., Dobos G., Cramer H. (2019). The use of mind-body medicine among US individuals with sleep problems: Analysis of the 2017 National Health Interview Survey data. Sleep Med..

[31] Lutz A., Jha A.P., Dunne J.D., Saron C.D. (2015). Investigating the Phenomenological Matrix of Mindfulness-Related Practices From a Neurocognitive Perspective. Am. Psychol..

[32] Gard T., Hölzel B.K., Lazar S.W. (2014). The potential effects of meditation on age-related cognitive decline: A systematic review. Ann. N. Y. Acad. Sci..

[33] Quintana-Hernández D.J., Miró-Barrachina M.T., Ibáñez-Fernández I.J., Pino A.S., Quintana-Montesdeoca M.P., Rodríguez-de Vera B., Morales-Casanova D., Pérez-Vieitez Mdel C., Rodríguez-García J., Bravo-Caraduje N. (2016). Mindfulness in the Maintenance of Cognitive Capacities in Alzheimer’s Disease: A Randomized Clinical Trial. J. Alzheimer’s Dis..

[34] Gong H., Ni C.-X., Liu Y.-Z., Zhang Y., Su W.-J., Lian Y.-J., Peng W., Jiang C.-L. (2016). Mindfulness meditation for insomnia: A meta-analysis of randomized controlled trials. J. Psychosom. Res..

[35] Ludwig D.S., Kabat-Zinn J. (2008). Mindfulness in Medicine. JAMA J. Am. Med. Assoc..

[36] Rusch H.L., Rosario M., Levison L.M., Olivera A., Livingston W.S., Wu T., Gill J.M. (2019). The effect of mindfulness meditation on sleep quality: A systematic review and meta-analysis of randomized controlled trials. Ann. N. Y. Acad. Sci..

[37] Britton W.B., Haynes P.L., Fridel K.W., Bootzin R.R. (2012). Mindfulness-based cognitive therapy improves polysomnographic and subjective sleep profiles in antidepressant users with Sleep complaints. Psychother. Psychosom..

[38] Ashton N.J., Hye A., Leckey C.A., Jones A.R., Gardner A., Elliott C., Wetherell J.L., Lenze E.J., Killick R., Marchant N.L. (2017). Plasma REST: A novel candidate biomarker of Alzheimer’s disease is modified by psychological intervention in an at-risk population. Transl. Psychiatry.

[39] Larouche E., Hudon C., Goulet S. (2015). Potential benefits of mindfulness-based interventions in mild cognitive impairment and Alzheimer’s disease: An interdisciplinary perspective. Behav. Brain Res..

[40] Malinowski P., Shalamanova L. (2017). Meditation and Cognitive Ageing: The Role of Mindfulness Meditation in Building Cognitive Reserve. J. Cogn. Enhanc..

[41] Shim M., Tilley J.L., Im S., Price K., Gonzalez A. (2021). A Systematic Review of Mindfulness-Based Interventions for Patients with Mild Cognitive Impairment or Dementia and Caregivers. J. Geriatr. Psychiatry Neurol..

[42] Chen T.-L., Chang S.-C., Hsieh H.-F., Huang C.-Y., Chuang J.-H., Wang H.-H. (2020). Effects of mindfulness-based stress reduction on sleep quality and mental health for insomnia patients: A meta-analysis. J. Psychosom. Res..

[43] Wang Y.-Y., Wang F., Zheng W., Zhang L., Ng C.H., Ungvari G.S., Xiang Y.-T. (2020). Mindfulness-Based Interventions for Insomnia: A Meta-Analysis of Randomized Controlled Trials. Behav. Sleep Med..

[44] Blackman J., Swirski M., Clynes J., Harding S., Leng Y., Coulthard E. (2021). Pharmacological and non-pharmacological interventions to enhance sleep in mild cognitive impairment and mild Alzheimer’s disease: A systematic review. J. Sleep Res..

[45] He X., Hao J., Song Y., Cao H., Chen Y., Yang H. (2023). Effectiveness of non-pharmacological interventions for sleep disturbances in people living with dementia: A systematic review and meta-analysis. Geriatr. Nurs..

[46] O’Caoimh R., Mannion H., Sezgin D., O’Donovan M.R., Liew A., Molloy D.W. (2019). Non-pharmacological treatments for sleep disturbance in mild cognitive impairment and dementia: A systematic review and meta-analysis. Maturitas.

[47] Wilfling D., Calo S., Dichter M.N., Meyer G., Möhler R., Köpke S. (2023). Non-pharmacological interventions for sleep disturbances in people with dementia. Cochrane Database Syst. Rev..

[48] Bentley T.G.K., Castillo D., Sadeghi N., Piber D., Carroll J., Olmstead R., Irwin M.R. (2022). Costs associated with treatment of insomnia in Alzheimer’s disease caregivers: A comparison of mindfulness meditation and cognitive behavioral therapy for insomnia. BMC Health Serv. Res..

[49] Arksey H., O’Malley L. (2005). Scoping studies: Towards a methodological framework. Int. J. Soc. Res..

[50] Munn Z., Peters M.D.J., Stern C., Tufanaru C., McArthur A., Aromataris E. (2018). Systematic review or scoping review? Guidance for authors when choosing between a systematic or scoping review approach. BMC Med. Res. Methodol..

[51] Peters M.D.J., Godfrey C.M., Khalil H., McInerney P., Parker D., Soares C.B. (2015). Guidance for conducting systematic scoping reviews. Int. J. Evid.-Based Healthc..

[52] Peters M.D.J., Godfrey C.M., McInerney P. (2015). Methodology for JBI Scoping Reviews. The Joanna Briggs Institute Reviewers’ Manual 2015.

[53] Tricco A.C., Lillie E., Zarin W., O’Brien K.K., Colquhoun H., Levac D., Moher D., Peters M.D.J., Horsley T., Weeks L. (2018). PRISMA extension for Scoping Reviews (PRISMA-ScR): Checklist and explanation. Ann. Intern. Med..

[54] McKeown S., Mir Z.M. (2021). Considerations for conducting systematic reviews: Evaluating the performance of different methods for de-duplicating references. Syst. Rev..

[55] van de Schoot R., de Bruin J., Schram R., Zahedi P., de Boer J., Weijdema F., Kramer B., Huijts M., Hoogerwerf M., Ferdinands G. (2021). An open source machine learning framework for efficient and transparent systematic reviews. Nat. Mach. Intell..

[56] van Dijk S.H.B., Brusse-Keizer M.G.J., Bucsán C.C., van der Palen J., Doggen C.J.M., Lenferink A. (2023). Artificial intelligence in systematic reviews: Promising when appropriately used. BMJ Open.

[57] Hong Q.N., Fàbregues S., Bartlett G., Boardman F., Cargo M., Dagenais P., Gagnon M.-P., Griffiths F., Nicolau B., O’Cathain A. (2018). The Mixed Methods Appraisal Tool (MMAT) Version 2018 for Information Professionals and Researchers. Educ. Inf..

[58] Innes K.E., Montgomery C., Selfe T.K., Wen S., Khalsa D.S., Flick M. (2021). Incorporating a usual care comparator into a study of meditation and music listening for older adults with subjective cognitive decline: A randomized feasibility trial. J. Alzheimer’s Dis. Rep..

[59] Innes K.E., Selfe T.K., Brundage K., Montgomery C., Wen S., Kandati S., Bowles H., Khalsa D.S., Huysmans Z. (2018). Effects of meditation and music-listening on blood biomarkers of cellular aging and Alzheimer’s Disease in adults with subjective cognitive decline: An exploratory randomized clinical trial. J. Alzheimer’s Dis..

[60] Innes K.E., Selfe T.K., Khalsa D.S., Kandati S. (2016). Effects of meditation versus music listening on perceived stress, mood, sleep, and quality of life in adults with early memory loss: A pilot randomized controlled trial. J. Alzheimer’s Dis..

[61] Cai Z.Z., Lin R., Wang X.X., Yan Y.J., Li H. (2022). Effects of mindfulness in patients with mild cognitive impairment with insomnia: A double-blind randomized controlled trial. Geriatr. Nurs..

[62] Giulietti M.V., Spatuzzi R., Fabbietti P., Vespa A. (2023). Effects of Mindfulness-Based Interventions (MBIs) in patients with early-stage Alzheimer’s Disease: A pilot study. Brain Sci..

[63] Paller K.A., Creery J.D., Florczak S.M., Weintraub S., Mesulam M.M., Reber P.J., Kiragu J., Rooks J., Safron A., Morhardt D. (2015). Benefits of mindfulness training for patients with progressive cognitive decline and their caregivers. Am. J. Alzheimer’s Dis. Other Dement..

[64] Kovach C.R., Evans C.R., Sattell L., Rosenau K., Gopalakrishnan S. (2018). Feasibility and pilot testing of a mindfulness intervention for frail older adults and individuals with dementia. Res. Gerontol. Nurs..

[65] Ware J.E., Kosinski M. (2001). Interpreting SF-36 Summary Health Measures: A Response. Qual. Life Res..

[66] McCoy C.E. (2017). Understanding the Intention-to-treat principle in randomized controlled trials. West. J. Emerg. Med..

[67] Rash J.A., Kavanagh V.A.J., Garland S.N. (2022). A meta-analysis of mindfulness-based therapies for insomnia and sleep disturbance moving toward processes of change. Sleep Med. Clin..

[68] Cassidy-Eagle E., Siebern A., Unti L., Glassman J., O’Hara R. (2018). Neuropsychological functioning in older adults with mild cognitive impairment and insomnia randomized to CBT-I or control group. Clin. Gerontol..

[69] He J., Chan S.H.W., Lin J., Tsang H.W.H. (2024). Integration of tai chi and repetitive transcranial magnetic stimulation for sleep disturbances in older adults: A pilot randomized controlled trial. Sleep Med..

[70] Huberty J.L., Green J., Puzia M.E., Larkey L., Laird B., Vranceanu A.-M., Vlisides-Henry R., Irwin M.R. (2021). Testing a mindfulness meditation mobile app for the treatment of sleep-related symptoms in adults with sleep disturbance: A randomized controlled trial. PLoS ONE.

[71] Johnson L.C.M., Aiello J.J., Jagtiani A., Moore K.N., Barber L., Gujral U.P., Johnson D.A. (2023). Feasibility, appropriateness, and acceptability of a mobile mindfulness meditation intervention to improve sleep quality among a racially/ethnically diverse population. Sleep Health.

[72] Stankeviciute L., Blackman J., Tort-Colet N., Fernández-Arcos A., Sánchez-Benavides G., Suárez-Calvet M., Iranzo Á., Molinuevo J.L., Gispert J.D., Coulthard E. (2024). Memory performance mediates subjective sleep quality associations with cerebrospinal fluid Alzheimer’s disease biomarker levels and hippocampal volume among individuals with mild cognitive symptoms. J. Sleep Res..

[73] Blackman J., Butters A., Oliver C., Coulthard E. (2023). Sleep Measurement in Mild Cognitive Impairment and Early Dementia: Is It Time for a Rethink?. Sleep.

